# Pragmatic applications of implementation science frameworks to regulatory science: an assessment of FDA Risk Evaluation and Mitigation Strategies (REMS) (2014–2018)

**DOI:** 10.1186/s12913-021-06808-3

**Published:** 2021-08-06

**Authors:** Linda Huynh, Gita A. Toyserkani, Elaine H. Morrato

**Affiliations:** 1 Food and Drug Administration, Silver Spring, MD USA; 2 Oak Ridge Institute for Science and Education (ORISE) Program, Oak Ridge, TN USA; 3 Parkinson School of Health Sciences and Public Health, Loyola University Chicago, Chicago, IL USA; 4 Colorado School of Public Health, University of Colorado Anschutz Medical Campus, Aurora, CO USA

**Keywords:** FDA, Drug safety, Risk Evaluation and Mitigation Strategies (REMS), Assessment, RE-AIM, PRECEDE-PROCEED, CFIR, Implementation science

## Abstract

**Background:**

A Risk Evaluation and Mitigation Strategy (REMS) is a drug safety program for certain medications with serious safety concerns required by the U.S. Food and Drug Administration (FDA) of manufacturers to implement to help ensure the benefits of the medication outweigh its risks. FDA is encouraging “the research community to develop novel methods for assessing REMS,” conveying the unmet need for a standardized evaluation method of these regulatory-mandated healthcare programs. The objective of this research is to evaluate FDA REMS assessment plans using established implementation science frameworks and identify opportunities for strengthening REMS evaluation.

**Methods:**

A content analysis was conducted of publicly available assessment plans for all REMS programs (*N* = 23) approved 1/1/2014–12/31/2018 for new drug applications (NDAs) and biologics license applications (BLAs) requiring FDA-mandated Elements to Assure Safe Use (ETASU). Blinded reviewers critically appraised REMS assessment measures (*n* = 674) using three established implementation science frameworks: RE-AIM (Reach, Effectiveness, Adoption, Implementation, Maintenance); PRECEDE-PROCEED (Predisposing, Reinforcing, and Enabling Constructs in Educational/Environmental Diagnosis and Evaluation – Policy, Regulatory, and Organizational Constructs in Educational and Environmental Development); and CFIR (Consolidated Framework for Implementation Research). Framework constructs were mapped to REMS Assessment categories as defined by FDA Guidance for Industry to evaluate congruence.

**Results:**

REMS assessment measures demonstrated strong congruence (> 90% mapping rate) with the evaluative constructs of RE-AIM, PRECEDE-PROCEED, and CFIR. Application of the frameworks revealed that REMS assessment measures heavily emphasize implementation and operations, focus less on health outcomes, and do not evaluate program context and design assumptions.

**Conclusions:**

Implementation science frameworks have utility for evaluating FDA-mandated drug safety programs including the selection of primary measures to determine whether REMS goals are being met and of secondary measures to evaluate contextual factors affecting REMS effectiveness in varying organizational settings.

**Supplementary Information:**

The online version contains supplementary material available at 10.1186/s12913-021-06808-3.

## Background

Risk Evaluation and Mitigation Strategies (REMS) are a drug safety program required by the US Food and Drug Administration (FDA) for certain medications with serious safety concerns to help ensure that the benefits of a medication outweigh its risks [1]. REMS are required when additional strategies beyond product labeling are needed to reduce the occurrence and/or severity of a specific risk to reinforce the medication’s safe use conditions and behaviors [2]. Between 2014 and 2018, approximately 4% of all drugs and biologics were approved on the condition of having a REMS [3, 4].

Table 1 presents a general REMS program overview. REMS programs address a specific drug-safety situation and risk mitigation goal, such as, preventing, decreasing the frequency or severity of the serious risk, and/or screening for the risk [5]. Pharmaceutical manufacturers (i.e., sponsors) are required to implement REMS programs based on FDA requirements and healthcare settings and providers need to adopt these requirements. REMS activities, as defined by FDA’s regulatory authority, can include complex multi-level interventions known as elements to assure safe use (ETASU). Some ETASU target healthcare professionals and healthcare settings, requiring them to be certified to administer and dispense the medication or restrict the dispensing of a medication to a certain setting. Other ETASU may also include monitoring of patients, enrollment of patients in a registry, or certain packaging and safe disposal technologies. Approximately 80% of currently active REMS include at least one ETASU [6]. In addition to ETASU, other REMS strategies include dissemination of information such as materials in patient-friendly language delivered to patients (Medication Guide, Patient Package Insert) and communication materials to healthcare providers (Communication Plan) [7, 8].

**Table 1:** REMS Program Overview

Goal	Stakeholders	REMS Intervention	Process Assessment	Outcome Assessment
▪ Drug safety risk assessment and mitigation	▪ FDA: regulatory requirements and guidance ▪ Pharmaceutical manufacturers: implementation and assessment requirements ▪ Health care settings/providers: adoption and risk mitigation delivery ▪ Patient/caregiver: program recipient	Intervention strategies, as specified by FDA REMS authority: ▪ Medication Guide (patient communication) ▪ Communication Plan (health care provider communication) ▪ Elements to Assure Safe Use • Certification or specialized training, prescriber • Certification or specialized training, pharmacy or other dispensing setting • Dispensing/drug administration requirement, limited settings • Dispensing/drug administration requirement, evidence of safe-use conditions being met • Patient, monitoring requirement • Patient, registry enrollment	REMS participation assessment category, as defined by FDA draft Guidance to Industry: ▪ Program Outreach and Communication ▪ Program Implementation and Operations	REMS outcome assessment category, as defined by FDA draft Guidance to Industry: ▪ Knowledge ▪ Safe Use Behavior ▪ Health Outcomes and/or Surrogate Health Outcomes Other impact considerations: ▪ Burden on the health care delivery system ▪ Barriers to patient access

Program assessment is required for all REMS approved for drugs or biologics under New Drug Applications (NDA) and Biologics License Applications (BLA) and encompasses program participation, outcome and impact measures. Sponsors are required to conduct assessments of each REMS program and provide them to the Agency at defined timetables to determine whether the REMS is meeting its risk mitigation goal(s) [1, 9]. These reports should provide information and data that are based on the REMS assessment plan, which is a list of metrics on outreach and communications, knowledge, safe use behaviors, implementation and operations, and health outcomes that drug sponsors need to address. While the statute requires a timetable for submission of an assessment of the REMS, it does not specify how those assessments should be conducted [10].

Since the REMS authorities went into effect in 2008, there has been increased focus on the standardization of REMS assessments. In 2013, the Office of Inspector General (OIG) report urged the FDA to “identify and implement reliable methods to assess the effectiveness of REMS.” [11] In that same year, FDA hosted a *Standardizing and Evaluating REMS Public Meeting* under its Prescription Drug User Fee Act (PDUFA) V commitments to obtain input on working towards “an evidence-based approach to assessing the effectiveness and burden of REMS” and “to identify best practices to incorporate into future REMS design, as well as appropriate ways to standardize REMS tools and integrate REMS into the health care delivery system.” [12] In January 2019, the draft guidance for industry on *REMS Assessment: Planning and Reporting* (henceforth referred to as the *Assessment Guidance)* calls upon “applicants and the research community to develop novel methods for assessing REMS.” [8].

Implementation science has been widely applied to health related research to support large-scale evaluations of evidence-based practices and organizational interventions in real-world settings [13]. FDA-mandated drug safety programs similarly seek to integrate processes into the larger healthcare system by targeting individual behaviors and organizational activities; implementation science can lend its theories, frameworks, and research to the more narrowly focused field of pharmaceutical regulation. Viewed through a regulatory risk management lens, implementation is analogous to risk mitigation. Implementation frameworks provide a visual display of a program to help guide one’s thinking and development of the program. The current landscape presents an opportune time to continue the progress made and borrow from the field of implementation science to advance the science of REMS assessment and the field of pharmaceutical risk management [14, 15]. Since 2014, Smith and Morrato have argued that minimal attention to frameworks in the design of risk minimization programs has hampered the identification of factors contributing to their success [14, 15].

Previous work in recent years have called for increased transparency and standardization of the evaluation of pharmaceutical risk minimization programs. Members of the International Society for Pharmacoepidemiology, Smith et al. used principles from public health intervention design and evaluation to develop a quality reporting checklist informed by a framework from program theory and process evaluation [16]. The resulting RIMES statement is usable for regulators and the pharmaceutical industry and emphasizes the utility of similar standardized reporting to design higher-quality risk minimization evaluation studies and ultimately improve the quality and effectiveness of risk minimization programs. More globally, the European Medicines Agency has continued publishing guidelines on risk minimization measures (RMMs) specific to evaluating the effectiveness of outcomes identified. These discuss qualitative and quantitative data sources and methodologies [17].

The purpose of this research is to evaluate three different implementation science frameworks and their applicability for REMS assessments. This work expands on previous research characterizing REMS assessment plans [18], by comparing and contrasting additional frameworks and by expanding the analysis to include Shared System REMS involving multiple application holders including generics (abbreviated new drug applications or ANDAs). The aim of this structured analysis is to advance the science of REMS assessment and promote the uptake of a scientific approach for program design and evaluation of global pharmaceutical risk management plans. Furthermore, this research has implications for the FDA to consider the application of implementation science frameworks when finalizing its REMS assessment guidance.

## Methods

### Selection of frameworks

Selection of eligible frameworks was done through a repository of dissemination and implementation frameworks found through the NIH’s Office of Disease Prevention at dissemination-implementation.org [19]. One member of the research team (LH) defined criteria for selecting frameworks with most effective applicability to REMS. Frameworks had to be in the fields of health, public health, or health services. Because REMS programs encompass risk mitigation strategies, frameworks were selected if they focused on either implementation alone or dissemination and implementation equally. Due to the nature of most REMS programs as multi-level interventions, frameworks also had to characterize the individual, organization, and/or community levels. To operationalize these frameworks into a REMS context, it was appropriate for them to score at least a ‘3’ out of 5 on construct flexibility, with a ‘1’ being broad or flexible in definition and a ‘5’ being detailed with step-by-step actions. Finally, as REMS are FDA-required programs, it was also appropriate to select frameworks that were United States-based.

Five frameworks met inclusion criteria, with two of them being derivations of another two. To select the most applicable frameworks for our research, we narrowed these five down to the top three that were most well-established and supported in the literature, each representing different schools of thought. The resulting frameworks are as follows: RE-AIM (Reach, Effectiveness, Adoption, Implementation, Maintenance) from implementation science, PRECEDE-PROCEED (Predisposing, Reinforcing Enabling, Construct in, Educational, Diagnosis and Evaluation – Policy, Regulatory, Organizational, Construct in, Educational and Environmental, Development) from health program planning and evaluation, and CFIR (Consolidated Framework for Implementation Research) from clinical quality improvement [20–22].

To identify current initiatives in the space intersecting implementation science and risk management, a literature review was conducted in January 2019 to search for articles relating REMS to dissemination and implementation science frameworks using three databases: PubMed, Web of Science, and EMBASE. The database searches were updated August 2020. We inputted search strings consisting of common implementation science and REMS terminology. Examples of these search strings include “risk evaluation and mitigation strategies” AND “implementation framework.” A more complete list of these terms can be found in Additional file 1. In January 2019, these searches produced no articles and only one abstract relating RE-AIM to a specific REMS program [23]. The updated August 2020 search produced one article [18].

### Application of frameworks to REMS assessment plans

A content analysis of REMS assessment plans was conducted for REMS programs approved by the FDA between 1/1/2014–12/31/2018 [24]. With the first REMS approved in 2008, this timeframe was selected to align with more current REMS approvals. REMS assessment plans were eligible if they were: (1) for a new drug application (NDA) or a biologics license application (BLA) and (2) included ETASU (Table 2**)**. Shared System REMS, which reflect multiple products, including generics, of the same class or molecular moiety under two or more sponsors [25] were included in the analysis. However, REMS comprised of only Communication Plans and/or Medication Guides were excluded from our analysis due to our focus on complex, multi-level, multi-system interventions. REMS assessment plans can be publicly accessed for individual programs through the REMS@FDA website, which link to the Drugs@FDA website, where each drug approval letter containing a list of metrics for the REMS can be found. The unit of analysis of this research focused on REMS assessment items as they are metrics for evaluating the performance of a REMS towards meeting its risk mitigation goals.

**Table 2:** Characteristics of selected REMS programs at time of original approval, active January 2019

Type	Drug (active ingredient)**	Year	ETASU***	Indication (Benefit)	Risk(s) requiring Risk Mitigation	Number of Assessment Items
ANDA	Clozapine	2015	A, B, D, E, F	treatment-resistant schizophrenia; reducing suicidal behavior in patients with schizophrenia or schizoaffective disorder	Neutropenia	22
	Alosetron	2016	A	severe diarrhea-prominent irritable bowel syndrome in women	Ischemic colitis and serious complications of constipation	10
	Sodium Oxybate	2017	A, B, D (MG)	treatment of cataplexy or excessive daytime sleepiness in narcolepsy	Serious adverse outcomes resulting from inappropriate prescribing, misuse, abuse, and diversion	71
	Vigabatrin	2017	A, B, D, E	treatment of refractory complex partial seizures in adults and infantile spasms	Vision loss	23
	Emtricitabine/Tenofovir Disoproxil Fumarate	2017	A	treatment of HIV-1 infection	Acquiring HIV-1 infection	8
BLA	Myalept (*metreleptin*)	2014	A, B, D	treat the complications of leptin deficiency in patients with congenital or acquired generalized lipodystrophy	Lymphoma & anti-metreleptin antibodies that neutralize endogenous leptin and/or Myalept	26
	Lemtrada (*alemtuzumab*)	2014	A, B, C, D (CP)	treatment of patients with relapsing forms of multiple sclerosis	Autoimmune conditions, infusion reactions, and malignancies	36
	Natpara (*parathyroid hormone*)	2015	A, B, D	an adjunct to calcium and vitamin D to control hypocalcemia in patients with hypoparathyroidism	Osteosarcoma	21
	Zinbryta (*daclizumab*)	2017	A, B, D, E, F (CP)	treatment of adult patients with relapsing forms of multiple sclerosis	Hepatic injury & immune mediated disorders	27
	Siliq (*brodalumab*)	2017	A, B, D	treatment of moderate to severe plaque psoriasis in adult patients who are candidates for systemic therapy or phototherapy and have failed to respond or have lost response to other systemic therapies	Suicidal ideation and behavior, including completed suicides	31
	Kymriah (*tisagenlecleucel*)	2017	B, C	treatment of: Pediatric and Young Adult Relapsed or Refractory (r/r) B-cell Acute Lymphoblastic Leukemia & Adult Relapsed or Refractory (r/r) Diffuse Large B-Cell Lymphoma	Cytokine release syndrome & neurological toxicities	21
	Yescarta (*axicabtagene ciloleucel*)	2017	B, C	treatment of adult patients with relapsed or refractory large B-cell lymphoma after two or more lines of systemic therapy	Cytokine release syndrome & neurological toxicities	21
	Palynziq (*pegvaliase-pqpz*)	2018	A, B, D	reduce blood phenylalanine concentrations in adult patients with phenylketonuria who have uncontrolled blood phenylalanine concentrations greater than 600 micromol/L on existing management	Anaphylaxis	31
	Ultomiris (*ravulizumab-cwvz*)	2018	A	treatment of adult patients with paroxysmal nocturnal hemoglobinuria	Meningococcal infections	10
NDA	Aveed (testosterone undecanoate)	2014	A, B, C	testosterone replacement therapy in adult males for conditions associated with a deficiency or absence of endogenous testosterone	Autoimmune conditions, infusion reactions, and malignancies	36
	Xyrem (*Sodium oxybate*)	2015	A, B, D (MG)	treatment of cataplexy or excessive daytime sleepiness in patients 7 years of age and older with narcolepsy	Serious adverse outcomes resulting from inappropriate prescribing, misuse, abuse, and diversion	57
	Ionsys (*fentanyl iontophoretic) transdermal system*)	2015	B, C	short-term management of acute postoperative pain severe enough to require an opioid analgesic in the hospital and for which alternative treatments are inadequate	Respiratory depression resulting from accidental exposure	29
	Addyi (*flibanserin*)	2015	A, B	treatment of premenopausal women with acquired, generalized hypoactive sexual desire disorder, as characterized by low sexual desire that causes marked distress or interpersonal difficulty	Hypotension and syncope due to interaction with alcohol	32
	Probuphine (*buprenorphine hydrochloride*)	2016	A, B, C, E (MG)	maintenance treatment of opioid dependence in patients who have achieved and sustained prolonged clinical stability on low-to-moderate doses of a transmucosal buprenorphine-containing product	Migration, protrusion, expulsion and nerve damage associated with insertion and removal & accidental overdose, misuse and abuse	22
	Sublocade (*buprenorphine extended-release*)	2017	B	treatment of moderate to severe opioid use disorder in patients who have initiated treatment with a transmucosal buprenorphine-containing product, followed by dose adjustment for a minimum of 7 days	Intravenous self-administration	20
	Jynarque (*tolvaptan*)	2018	A, B, D, E, F (CP)	slow kidney function decline in adults at risk of rapidly progressing autosomal dominant polycystic kidney disease	Liver injury	42
	Tegsedi (*Inotersen*)	2018	A, B, D, E, F	treatment of polyneuropathy of hereditary transthyretin-mediated amyloidosis in adults	Bleeding with thrombocytopenia & glomerulonephritis	57
	Dsuvia (*sufentanil*)	2018	B, C	use in adults in certified medically supervised healthcare settings for the management of acute pain severe enough to require an opioid analgesic and for which alternative treatments are inadequate	Respiratory depression from accidental exposure	35

ANDA, Abbreviated New Drug Application; BLA, Biologic License Application; NDA, New Drug Application; ETASU, Elements to Assure Safe Use; CP, Communication Plan; MG, Medication Guide

** REMS programs were selected January 2019

***ETASU A, training or certification of prescribers; ETASU B, training or certification of dispensers; ETASU C, dispensing/administering the drug in certain settings; ETASU D, requiring evidence or documentation of safe use conditions; ETASU E, monitoring of patients; ETASU F, enrolling patients in a registry

Data source: Food and Drug Administration. Approved Risk Evaluation and Mitigation Strategies (REMS). Available from: https://www.accessdata.fda.gov/scripts/cder/rems/index.cfm (REMS@FDA). Also available from Drugs@FDA

Starting with the RE-AIM framework, three reviewers (LH, GT, EM) created construct definitions applicable to REMS assessments by adapting from RE-AIM dimensions defined by the framework (Table 3). After adjudicating these applications for three randomly selected plans (IRR = 75%), the review team then refined the definitions accordingly. Two reviewers (LH and GT) categorized each assessment item (*n* = 674) for the remaining 20 plans. This adaptation, adjudication, and refinement process was repeated for PRECEDE-PROCEED (IRR = 79%) and CFIR (IRR = 84%) (Tables 4, 5 and 6).

**Table 3:** Adaptation of RE-AIM dimensions as applied to REMS assessment measures and Assessment Guidance categories

RE-AIM Dimension	General Description*	Description as Applied to REMS Assessments**	Assessment Guidance Category	Definitions of Assessment Guidance Category
**R**each	Reach refers to the absolute number, proportion, and representativeness of individuals who are willing to participate in a given initiative, intervention, or program	**Patient (individual level)** • Number of patients treated or enrolled (numerator) • Proportion of eligible patients (“valid denominator” given the drug’s indicated use) treated or enrolled • Characteristics of patients treated or enrolled compared with nonparticipants – representativeness	Outreach and Communications	Measures of the extent to which the REMS materials reached the intended stakeholders
**E**ffectiveness	Effectiveness refers to the impact of an intervention on important outcomes, including potential negative effects, quality of life, and economic outcomes	**Patient (individual level)** • Knowledge-Attitudes; Process-Behavior; Health Outcomes and/or Surrogates • Positive and negative (unintended) impacts; observed vs. expected rates of effectiveness • Heterogeneity (variability) of effect across different subpopulations	Safe Use Behaviors and Knowledge Health Outcomes	Measures of the extent to which safe use conditions are being adopted or followed, or of stakeholders’ knowledge about the REMS-related risk or knowledge of any safe use conditions Measures of the safety-related health outcome of interest or a surrogate of a health outcome
**A**doption	Adoption refers to the absolute number, proportion, and representativeness of settings and intervention agents (people who deliver the program) who are willing to initiate the program	**Health Care System (setting level)** • Number of practices, clinics, hospitals or pharmacies certified or enrolled (numerator) • Proportion of eligible practices, clinics, hospitals or pharmacies (“valid denominator” given the drug’s indicated use) certified or enrolled • Characteristics of practices, clinics, hospitals or pharmacies certified or enrolled compared with non-adopters – representativeness **Health Care Provider (agent level)** • Number of prescribers and/or pharmacists certified or enrolled (numerator) • Proportion of eligible prescribers and/or pharmacists (“valid denominator” given the drug’s indicated use) certified or enrolled • Characteristics of prescribers and/or pharmacists certified or enrolled compared with non-adopters – representativeness	Outreach and Communications	Measures of the extent to which the REMS materials reached the intended stakeholders
**I**mplementation	At the setting level, implementation refers to the intervention agents’ fidelity to the various elements of an intervention’s protocol, including consistency of delivery as intended and the time and cost of the intervention Implementation elements include: implementation fidelity, adaptation, and cost of intervention At the agent level, implementation refers to the clients’ use of the intervention strategies	**Health Care System (setting level)** • Percent of targeted groups who were sent, received REMS information and/or training (by mode and frequency of distribution) • Curriculum consistency – fidelity and adaptation over time (by training modality) • Extent of completed, successful training and/or certification in the program • Incremental costs and resources required (fixed and variable) for REMS participation • Heterogeneity (variability) of implementation across different settings	Implementation and Operations	Measures of the extent to which the intended stakeholders are participating in the program, how effectively the REMS program is being implemented and any unintended consequences such as patient access or burden to the healthcare system
		**Health Care Provider (agent level)** • Educational effectiveness measured by: knowledge-attitudes, behavioral intention for safe use processes and procedures, observed behavior-compliance • Heterogeneity (variability) of implementation across different settings and/or provider characteristics	Safe Use Behaviors and Knowledge	Measures of the extent to which safe use conditions are being adopted or followed, or of stakeholders’ knowledge about the REMS-related risk or knowledge of any safe use conditions
**M**aintenance	At the setting level, maintenance reflects the extent to which the program or processes become institutionalized or sustained as part of routine practice over time	**Health Care System (setting level)** • Cumulative real-world evidence of the integration of REMS processes and procedures into state and institutional policies, treatment guidelines, insurance requirements	Not included	Not applicable
	At the agent or individual level, maintenance reflects the extent to which practices become a stable part of the behavioral repertoire of the individual	**Health Care Provider (agent level) & Patient (individual level)** • Cumulative evidence over time to include: durability of knowledge; compliance with REMS processes and procedures; attrition rate (from the program); heterogeneity (variability) of attrition by subgroups, unintended outcomes, e.g., access or burden issues	Not included	Not applicable

Not included: no appropriate category from the Assessment Guidance. Not applicable: not currently assessed in the REMS program to have application

*Defined in Gaglio B SJ, Glasgow RE. The RE-AIM Framework: A Systematic Review of Use Over Time. American Journal of Public Health (AJPH) (2013) 103(6):38–46

**Informed by the National Cancer Institute. RE-AIM Scoring Instrument [updated 09/04/2013]. Available from: https://rtips.cancer.gov/rtips/reAim.do

**Table 4:** Adaptation of PRECEDE-PROCEED phases as applied to REMS assessment measures and Assessment Guidance categories. **P**redisposing, **R**einforcing, and **E**nabling **C**onstructs in **E**ducational / Ecological **D**iagnosis and **E**valuation (PRECEDE Structure)

PRECEDE Phase	General Description	Description as Applied to REMS Assessments	Assessment Guidance Category	Definitions of Assessment Guidance Categories
1. Social Assessment	Determine what the target population wants and the ultimate desired result. Identify specific health problems that may contribute to the target population’s quality of life, social goals, or problems; Diagnose severity of the problem as perceived by the target population	Summarize the benefit-risk profile of the drug and unmet medical need Identify patient health and treatment preferences for the target patient group Determine if patients are willing to take on risks associated with REMS medications in the context of treatment benefits, ensuring that they are sufficiently informed to share decision making and are confident in their ability to comply with REMS.	Not included	Not applicable
2. Epidemiological Assessment	Identify the prevalence, health determinants, and burden of the identified problems; Set priorities and goals	Benefit-risk assessment that a REMS is necessary to ensure the benefits outweigh the risks Identify the gap in knowledge or aspect of human behavior that leads to risk (e.g. alcohol use resulting in an interaction with the drug) Epidemiological data of the risks that the REMS is intended to address Epidemiological data on the target population REMS goal presented in a “SMART” format – the overall, safety-related health outcome(s) that the REMS is designed to achieve	Not included	Not applicable
3. Educational and Ecological Assessment	Categorize the predisposing, enabling, or reinforcing factors most likely to result in behavior change	Identify workflow preferences for the target healthcare provider Intermediate, measurable REMS objectives presented in a “SMART” format Determine which REMS materials and Participant Requirements would be most inducive to achieving goal. Health literacy & self-efficacy (ability to do what is required)	Not included	Not applicable
4. Administrative and Policy Assessment and Intervention Alignment	Identify administrative, organizational, and policy factors required for development and implementation of the intervention as well as resources and barriers	Identify clinical workflow preferences for the target healthcare settings Determine the REMS interventions e.g., ETASU to achieve objectives or other Applicant Requirements Clinical/medical practice guidelines	Not included	Not applicable

**Table 5:** **P**olicy, **R**egulatory, and **O**rganizational **C**onstructs in **E**ducational and **E**nvironmental **D**evelopment (PROCEED Structure)

PROCEED Phase	General Description	Description as applied to REMS Assessments	Assessment Guidance Category	Definitions of Assessment Guidance Categories
5. Implementation	Design intervention; Assess availability of resources; Implement program	A. REMS Design (PRECEDE assumptions): • Alignment with patient preferences • Alignment with healthcare system and clinical workflow norms • Alignment with health insurance requirements and medical practice guidelines	Not included	Not applicable
		B. Availability of Resources: • Capacity and capability of the healthcare personnel to fulfill REMS requirements (human resources) • Availability (costs) of medical supplies and facilities necessary to fulfill REMS requirements (physical resources)	Not included	Not applicable
		C. Program Outreach and Communication: • Date of product or REMS launch • Date communication materials distributed (by mode and frequency, including website access) • Dates of training programs (by mode and frequency)	Program Outreach and Communication	Measures of the extent to which the REMS materials reached the intended stakeholders
6. Process Evaluation	Determine whether planned program put into action has been successfully implemented in target audience; Ongoing evaluation and modification of program components	A. Program Implementation *At the Health Care Setting-, Health Care Provider- and Patient-Level* • Number successfully certified, enrolled or receiving the medication; proportion of target audience certified or enrolled. • Characteristics of those certified, enrolled or receiving the medication compared with non-adopters in the same target audience – representativeness • Unenrollment (voluntary) of health care providers (and reasons)	Program Implementation and Operations	Measures of the extent to which the intended stakeholders are participating in the program, how effectively the REMS program is being implemented and any unintended consequences such as patient access or burden to the healthcare system
		B. Quality Process Improvement/System-Level Evaluation (by risk mitigation strategy) • Unintended interruptions and/or delays • Training curriculum fidelity and adaptation (by training modality) • Audit findings and corrective actions, including analysis of Call Center complaints and dispensing systems • Number (type) of REMS modifications	Program Implementation and Operations	Measures of the extent to which the intended stakeholders are participating in the program, how effectively the REMS program is being implemented and any unintended consequences such as patient access or burden to the healthcare system
7. Impact Evaluation	Determine effectiveness and efficiency of program in terms of intermediate objectives and change in predisposing, enabling, and reinforcing factors (immediate observable effects of the program); examines impact of program intervention on environmental and behavioral factors identified in PRECEDE phase of intervention design	A. Effectiveness: Knowledge and Skill *At the Health Care Provider- and Patient-Level* • Effectiveness of certification training • Knowledge and awareness of key risk messages	Knowledge	Measures of the extent of stakeholders’ knowledge about the REMS-related risk or knowledge of any safe use conditions that are needed in order to mitigate the risk
		B. Effectiveness: Compliance *At the Health Care Provider- and Patient-Level* • Behavioral intention (self-reported) to take appropriate risk mitigation or safe use behaviors • Risk mitigation and safe use behaviors (observed) • Decertification (required) of health care provider (and reasons)	Evaluation of Safe Use Conditions	Measures of the extent to which safe use conditions are being adopted or followed
		C. Efficiency • Evidence of integration into clinical workflows and perceived burden given health care professional preferences • Patient access and perceived burden given patient preferences • Unenrollment (voluntary) of patients (due to burden) • Clinical efficiency (e.g., Number Needed to Screen to mitigate risk)	Program Implementation and Operations	Measures of the extent to which the intended stakeholders are participating in the program, how effectively the REMS program is being implemented and any unintended consequences such as patient access or burden to the healthcare system
		D. Effectiveness: Environmental Factors • Change in predisposing, reinforcing, and enabling behavioral factors • Administrative and policy factors (e.g., treatment guidelines and insurance prescribing requirements)	Not included	Not applicable
8. Outcome Evaluation	Evaluate effectiveness of program implementation on outcomes identified in PRECEDE phase of intervention design and how health status has changed overall	o Mitigating the health risks (or surrogate outcomes) as stated in the REMS Goals o Rates of expected and unexpected adverse events related to REMS implementation o Unenrollment of patients (due to safety outcomes being targeted by the REMS program)	Health Outcomes and/or Surrogates of Health Outcomes Adverse Event Surveillance	Measures of the safety-related health outcome of interest or a surrogate of a health outcome

Not included: no appropriate category from the Assessment Guidance. Not applicable: not currently assessed in the REMS program to have application

**Table 6:** Adaptation of CFIR constructs as applied to REMS assessment measures and Assessment Guidance categories

CFIR construct	Short description	Description as Applied to REMS Assessments	Assessment Guidance Category	Definitions of Assessment Guidance Category
**I. INTERVENTION CHARACTERISTICS**				
A. Intervention Source	Perception of key stakeholders about whether the intervention is externally or internally developed.	Perception of key stakeholders’ about whether the REMS program is primarily developed externally (FDA/sponsor) or internally.	Not included	Not applicable
B. Evidence Strength & Quality	Stakeholders’ perceptions of the quality and validity of evidence supporting the belief that the intervention will have desired outcomes.	Stakeholders’ perception of the quality and validity of evidence supporting the belief that the program would achieve REMS goals.	Not included	Not applicable
C. Relative Advantage	Stakeholders’ perception of the advantage of implementing the intervention versus an alternative solution.	Stakeholders’ perception of the advantage of implementing the REMS versus an alternative solution, or not receiving the access to the product at all.	Not included	Not applicable
D. Adaptability	The degree to which an intervention can be adapted, tailored, refined, or reinvented to meet local needs.	The degree to which REMS requirements can be adapted, tailored, and refined to meet local needs and ease program implementation.	Not included	Not applicable
E. Trialability	The ability to test the intervention on a small scale in the organization, and to be able to reverse course (undo implementation) if warranted.	The degree to which the REMS program can be tested on a local scale or at specified points of care prior to implementation on a national level, or through a modification later in the program.	Not included	Not applicable
F. Complexity	Perceived difficulty of implementation, reflected by duration, scope, radicalness, disruptiveness, centrality, and intricacy and number of steps required to implement.	Perceived difficulty of REMS implementation, reflected by duration, scope, radicalness, disruptiveness, centrality, intricacy, and number of steps required to implement.	Not included	Not applicable
G. Design Quality & Packaging	Perceived excellence in how the intervention is bundled, presented, and assembled.	Perceived excellence of how the REMS program is bundled, presented, and assembled (i.e. quality of design and execution).	Not included	Not applicable
H. Cost	Costs of the intervention and costs associated with implementing the intervention including investment, supply, and opportunity costs.	Costs of the REMS program and costs associated with implementing the program including investment, supply, and opportunity costs.	Not included	Not applicable
**II. OUTER SETTING**				
A. Patient Needs & Resources	The extent to which patient needs, as well as barriers and facilitators to meet those needs, are accurately known and addressed by the organization.	The extent to which the REMS program is patient-focused and that patient needs, as well as barriers and facilitators to meet those needs, are accurately known and addressed by the sponsor.	Not included	Not applicable
B. Cosmopolitanism	The degree to which an organization is networked with other external organizations.	The extent and quality to which stakeholders are networked within the broader healthcare system to more quickly implement practices.	Not included	Not applicable
C. Peer Pressure	Mimetic or competitive pressure to implement an intervention; typically because most or other key peer or competing organizations have already implemented or are in a bid for a competitive edge.	Competitive pressure for healthcare providers to enroll in a REMS program due to the enrollment of other providers or those in the medical use process.	Not included	Not applicable
D. External Policy & Incentives	A broad construct that includes external strategies to spread interventions, including policy and regulations (governmental or other central entity), external mandates, recommendations and guidelines, pay-for-performance, collaboratives, and public or benchmark reporting.	Adoption encouraged by clinical care guidelines, reimbursement systems, and incentives such as pay-for-performance, and benchmark reporting.	Not included	Not applicable
**III. INNER SETTING**				
A. Structural Characteristics	The social architecture, age, maturity, and size of an organization.	The effects of the point of care’s size, degree of vertical integration, number of departments, number of units/departments, and degree of specialization on the implementation of individual REMS programs.	Not included	Not applicable
B. Networks & Communications	The nature and quality of webs of social networks and the nature and quality of formal and informal communications between sponsors and their vendors.	The strength of formal and informal communications, networking, and relationships between sponsors, vendors, and points of care and their effects on the adoption of the REMS program and understanding of its goals.	Not included	Not applicable
C. Culture	Norms, values, and basic assumptions of a given organization.	The norms, values, and basic assumptions about risk management and the REMS program at the point of care and the extent of how relatively stable, subconscious, and socially constructed these are.	Not included	Not applicable
D. Implementation Climate	The absorptive capacity for change, shared receptivity of involved individuals to an intervention, and the extent to which use of that intervention will be rewarded, supported, and expected within their organization.	The absorptive capacity for change, shared receptivity of involved individuals to the program, and the extent to which use of the REMS will be rewarded, supported, and expected through policies, procedures, and systems within the points of care.	Not included	Not applicable
E. Readiness for Implementation	Tangible and immediate indicators of organizational commitment to its decision to implement an intervention.	Tangible and immediate indicators of stakeholders’ readiness for adoption of the REMS in terms of setting, culture, leadership, and evaluation.	Not included	Not applicable
**IV. CHARACTERISTICS OF INDIVIDUALS**				
A. Knowledge & Beliefs about the Intervention	Individuals’ attitudes toward and value placed on the intervention as well as familiarity with facts, truths, and principles related to the intervention.	Participants’ attitudes toward and value placed on the REMS as well as familiarity with facts, truth, and principles related to the program, including sufficient knowledge of the necessity for and skill of executing the REMS program.	Knowledge	Measures of the extent of stakeholders’ knowledge about the REMS-related risk or knowledge of any safe use conditions that are needed in order to mitigate the risk
B. Self-efficacy	Individual belief in their own capabilities to execute courses of action to achieve implementation goals.	Participants’ belief in their own capabilities to execute courses of action to achieve REMS goals.	Not included	Not applicable
C. Individual Stage of Change	Characterization of the phase an individual is in, as he or she progresses toward skilled, enthusiastic, and sustained use of the intervention.	Characterization of the phase a participant is in and additional strategies necessary for the skilled and enthusiastic maintenance of behavior.	Safe Use Behaviors	Measures of the extent to which safe use conditions are being adopted or followed
D. Individual Identification with Organization	A broad construct related to how individuals perceive the organization, and their relationship and degree of commitment with that organization.	A broad construct related to how participants perceive the REMS and their willingness to fully engage due to their degree of commitment with the sponsor.	Not included	Not applicable
E. Other Personal Attributes	A broad construct to include other personal traits such as tolerance of ambiguity, intellectual ability, motivation, values, competence, capacity, and learning style.	A broad construct to include other personal traits such as participants’ locus of control and other psychological concepts related to REMS implementation.	Not included	Not applicable
**V. PROCESS**				
A. Planning	The degree to which a scheme or method of behavior and tasks for implementing an intervention are developed in advance, and the quality of those schemes or methods.	The degree to which a scheme or method of behavior and tasks for implementing the REMS are developed in advance, and the quality of the evidence supporting those steps to promote effective implementation.	Not included	Not applicable
B. Engaging	Attracting and involving appropriate individuals in the implementation and use of the intervention through a combined strategy of social marketing, education, role modeling, training, and other similar activities.	Carefully and thoughtfully attracting involving appropriate representatives from each stakeholder group in the implementation of the REMS program through a combined strategy of social marketing, education, role modeling, training, and other similar activities to meet participants’ needs.	Program Outreach and Communication	Measures of the extent to which the REMS materials reached the intended stakeholders
C. Executing	Carrying out or accomplishing the implementation according to plan.	Carrying out or accomplishing the REMS program according to plan (descriptive).	Program Implementation and Operations	Measures of the extent to which the intended stakeholders are participating in the program, how effectively the REMS program is being implemented and any unintended consequences such as patient access or burden to the healthcare system
D. Reflecting & Evaluating	Quantitative and qualitative feedback about the progress and quality of implementation accompanied with regular personal and team debriefing about progress and experience.	Quantitative and qualitative feedback (evaluative) about the progress and quality REMS implementation accompanied with regular personal and team debriefing about progress and experience.	Program Implementation and Operations	Measures of the extent to which the intended stakeholders are participating in the program, how effectively the REMS program is being implemented and any unintended consequences such as patient access or burden to the healthcare system

Not included: no appropriate category from the Assessment Guidance. Not applicable: not currently assessed in the REMS program to have application

Shortly following the initiation of this research, the draft Assessment Guidance was published in January 2019 [8]. This draft Assessment Guidance outlined five categories that were intended to capture REMS program outcomes and processes. To evaluate the utility of the three frameworks on REMS assessments, these RE-AIM dimensions, PRECEDE-PROCEED phases, and CFIR constructs were then mapped to the Assessment Guidance categories: Program Outreach and Communication, Program Implementation and Operations, Knowledge, Safe Use Behaviors, and Health Outcomes and/or Surrogates of Health Outcomes (Additional file 2). *For simplicity, the dimensions of RE-AIM, phases of PRECEDE-PROCEED, and constructs of CFIR will be collectively referred to as “constructs” hereafter.* Because REMS programs are focused on achieving its goals through information, education, and/or reinforcement of actions, it was appropriate for us to combine knowledge and safe use behaviors into one assessment category to map to the constructs.

Results were reported as aggregate summary statistics for the frequency distribution across all programs and the number of assessment measures per Assessment Guidance category. Finally, sensitivity analysis was performed to examine qualitative differences by type of application (e.g., drug vs. biologic), type of ETASU, and trends over time. A subgroup analysis for Shared Systems was done in particular. For each framework, descriptive statistics were calculated to determine the proportions each construct was represented per REMS program. Each construct was analyzed for the number of REMS programs addressing the construct, the median number and range of measures representing that construct per REMS program, and the number of measures representing the construct across all programs. Additionally, each assessment item was assessed for inclusion of the Assessment Guidance categories using the mapping of each frameworks’ constructs.

## Results

A total of 23 REMS programs consisting of nine BLAs, nine NDAs, and five Shared Systems were selected for analysis based on the eligibility criteria (Additional file 3**)**. Characteristics of these programs at the time of their original REMS approval can be found in Table 2. Programs requiring a REMS varied by indication, including but not limited to B-cell lymphoma, acute pain requiring an opioid analgesic, and multiple sclerosis. Likewise, the risks intended to be mitigated by the REMS varied widely from neurological toxicities to respiratory depression and death. The number of REMS programs by year ranged from three in 2016 and 2018 to seven in 2017. Assessment measures per REMS program ranged from 8 to 71, for a total of 674 assessment measures across the 23 programs.

Subgroup analysis of Shared System REMS reveals they account for the minimum and maximum values of the number of assessment measures per REMS program, with emtricitabine tenofovir disoproxil fumarate containing 8 measures and sodium oxybate containing 71 measures. While the fewest number of assessment measures were associated with programs containing only ETASU A, no other associations can be made between ETASUs and assessment measures. REMS approval for Shared Systems mostly occurred in the latter years, with three programs approved in 2017 and one each in 2015 and 2016. This is reasonable because Shared System REMS reflect sustaining programs requiring multiple sponsors of the reference listed drug and ANDA to work together on the design and development of the REMS, a process requiring a substantial amount of time.

### Insights from the application of RE-AIM to REMS

From the participating pool of stakeholders comprised of patients, healthcare providers, pharmacies, wholesalers/distributors, and healthcare setting, defining RE-AIM constructs for application to REMS required the agent and recipient of the program to be identified. Recipients refer to patients or other populations who are targeted to benefit from the program outcomes produced, while agents are defined as people who deliver the program. Others have applied effectiveness to providers and the healthcare setting as well as implementation to patients [23, 26]. For our analysis, we defined patients as the recipients of the REMS program and providers, pharmacies, and wholesalers as the agents. Subsequently, the Reach and Effectiveness constructs applied to patients, Adoption and Implementation to providers/pharmacies/wholesalers, and Maintenance to any participant.

REMS assessment measures demonstrated the strongest congruence with the RE-AIM framework. All five RE-AIM constructs were represented with REMS assessment measures. Of 674 assessment measures across the 23 programs, only 4 measures (0.6%) could not be mapped to a single RE-AIM construct because either the intent of the assessment item was unclear or there were multiple intents of the assessment item, making it categorizable into multiple constructs. For example, we defined Reach to refer to the number of patients eligible to receive the drug who participate in the program and Adoption to refer to the number of agents (e.g., prescribers) involved in adopting and implementing the program (Table 3). Therefore, items assessing the number of prescriptions in compliance with REMS requirements were too ambiguous to classify to a single REMS domain because prescriptions could either reflect prescriber-compliant participation in the program through prescriber certification (Adoption) or the number of patients who ultimately received the medication (Reach).

Of 23 total REMS assessment plans, 19 (82.6%) contained measures assessing Reach, with the median number of Reach assessment measures per assessment plan being 2 (range 0–7). For Effectiveness, 20 (87.0%) REMS assessment plans included this construct, with the median number of assessment measures per plan being 2 (range 0–9). All 23 (100%) REMS assessment plans measured Adoption, with the median number of assessment measures per plan being 3 (range 0–7). Similarly, all 23 (100%) assessment plans also measured Implementation, however with a median number of assessment measures per plan being 18 (range 4–52). Finally, only 9 of 23 programs (39.1%) contained assessment measures of Maintenance, with the median number of assessment measures per plan being 0 (range 0–1).

Similarly, the median number of Reach assessment measures per Shared System assessment plan was also 2 (range 0–4). Notably, the Shared System REMS for emtricitabine tenofovir disoproxil fumarate contained the highest proportion of measures assessing Effectiveness, with this construct accounting for 37.5% of all assessment items. Adoption was similarly assessed among the Shared System REMS with the median number of assessment measures being 1 (range 1–5). The assessment of Implementation measures among Shared System REMS was comparable to that of other REMS programs, with the median number being 14 (range 4–52). Similar to the scarcity of other programs, only 1 Shared System REMS contained assessment measures of Maintenance, with the median number of assessment measures per plan being 0 (range 0–1). Shared System REMS shared the median number of Maintenance assessment measures compared to other programs, with this figure being 0 (range 0–1).

**Fig. 1:**
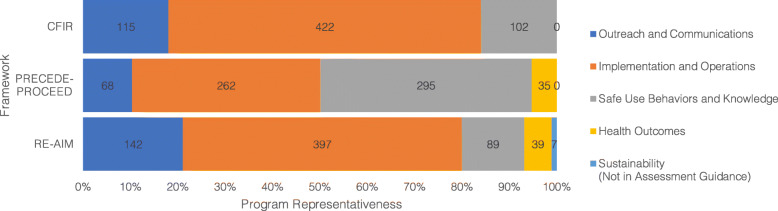
Number of REMS assessment measures (n = 674) across programs (N = 23) mapped to the Assessment Guidance by framework. CFIR, Consolidated Framework for Implementation Research; PRECEDE-PROCEED, Predisposing, Reinforcing Enabling, Construct in, Educational, Diagnosis and Evaluation – Policy, Regulatory, Organizational, Construct in, Educational and Environmental, Development; RE-AIM, Reach, Effectiveness, Adoption, Implementation, Maintenance

Categorizing the REMS assessment measures to the Assessment Guidance was also a feasible task (Fig. 1). Measures of Reach and Adoption were categorizable to Outreach and Communications, depending on whether the patient or provider was targeted. Those of Effectiveness were mapped to Health Outcomes if the item assessed adverse events or health risks and to Safe Use Behaviors and Knowledge if the item assessed patients’ understanding of the risks. Similarly, REMS assessment measures falling into the RE-AIM Implementation construct were categorizable to the Assessment Guidance’s Implementation and Operations category if they were process measures and into Safe Use Behaviors and Knowledge if they assessed providers/pharmacists’ understanding of the risks. Finally, although the Assessment Guidance does not include Maintenance as a category, some programs included a measure of Maintenance on their assessment plans.

### Insights from the application of PRECEDE-PROCEED to REMS

As a health program planning and evaluation model, PRECEDE-PROCEED provides structures for both specifying objectives and baselines before the intervention as well as for monitoring and continuous quality improvement after the intervention. However, as REMS include implementation and evaluation aspects, we did not apply PRECEDE and only defined the PROCEED constructs as applicable to REMS. Instead, we interpreted the “design intervention” step of the Implementation construct to assume that design assumptions from the PRECEDE constructs are met. This aligns to the nature of the PRECEDE-PROCEED framework, wherein change begins with the outcome, and the process moves backward logically to achieve the desired result in a formative process. Process Evaluation mapped to REMS assessment measures attributable to “the system,” while Impact Evaluation mapped measures relating to “the individual.” For constructs consisting of multiple components in the original definition, we divided these up into subconstructs when applying the definitions to REMS.

PRECEDE-PROCEED exemplified strong congruence to REMS assessment plans, with seven assessment measures not represented by the framework, as stakeholder engagement is not accessed in the PROCEED constructs. Therefore this 1% of REMS assessment measures that was not mapped to PRECEDE-PROCEED was related to antecedent outreach factors, such as sources of distribution lists. Not only were all PROCEED constructs represented by REMS assessment measures, but so were all subconstructs the research team developed (Tables 4 and 5).

Of 23 total REMS assessment plans, 18 (78.3%) contained measures assessing Implementation, with the median number of Implementation assessment measures per assessment plan being 4 (range 0–6). For Process Evaluation, all 23 (100%) REMS assessment plans included this construct, with the median number of assessment measures per plan being 12 (range 1–26). All 23 (100%) REMS assessment plans measured Impact Evaluation, with the median number of assessment measures per plan being 10 (range 3–34). Finally, 12 (52%) assessment plans measured Outcome Evaluation, with a median number of assessment measures per plan being 1 (range 0–6).

For Shared System REMS, the median number of Implementation assessment measures per plan was 1 (range 0–5). The median number of Process Evaluation measures per assessment plan was 10 (range 1–26). Notably, the Shared System REMS for emtricitabine tenofovir disoproxil fumarate contained the highest proportion of measures assessing Impact Evaluation, with this construct accounting for 87.5% of all its assessment measures and the median number of assessment measures per plan being 9 (4–34). Finally, only 2 Shared System REMS contained assessment measures of Outcome Evaluation, with the median number of Outcome Evaluation measures per plan mirroring that of the larger group at 0 (range 0–6).

Of the three frameworks, assessment items mapped to PRECEDE-PROCEED were most evenly distributed across the constructs. (Fig. 1). PROCEED constructs mapped most directly with those of the assessment guidance, with Implementation to Outreach and Communications, Process Evaluation to Implementation and Operations, Impact Evaluation to Safe Use Behaviors and Knowledge, and Outcome Evaluation to Health Outcomes.

### Insights from the application of CFIR to REMS

Developed out of 20 sources including the Diffusion of Innovations Theory, CFIR has been used for quality improvement [21]. Designed to provide a menu of constructs to be adaptable to a variety of applications, only the Characteristics of Individuals and Process constructs were most appropriate to REMS. Specifically, only the Knowledge and Beliefs about the Intervention and Individual Stage of Change under the Characteristics of Individuals construct, and the Engaging, Executing, and Reflecting and Evaluating under the Process constructs were applicable to REMS.

It is critical to note that CFIR is evidence-based, assuming the intervention is effective, and therefore does not measure outcomes. Given this, 5% of REMS assessment measures (34 of 674) did not map to the framework because they were outcome measures. Due to CFIR’s multilevel nature, it was important to define the “Inner Setting” to refer to individual sites of specific REMS programs, and “Outer Setting” to refer to the broader healthcare system inclusive of patients. A criticism of CFIR is the fact that its “combined breadth and depth is not always feasible for implementation,” [27]; however, its design for clinical quality improvement meant that it was easily customizable to a variety of REMS situations across diverse settings. In this context, “Characteristics of Individuals” was determined to represent any stakeholder (e.g. patient or provider) within that setting.

Of 23 total REMS assessment plans, 1 (4.4%) contained measures assessing Intervention Characteristics (Table 6), with the median number of Intervention Characteristics assessment measures per assessment plan being 2 (range 0–7). None of the REMS assessment measures included constructs that relate to Outer Setting and Inner Setting. Characteristics of Individuals were measured in 21 REMS assessment plans (91%), with the median number of assessment measures per plan being 18 (range 0–8) Finally, all 23 (100%) assessment plans measured Process, with a median number of assessment measures per plan being 20 (range 1–57).

Among Shared System REMS, the median number of Intervention Characteristics assessment measures per plan was 0 (range 0–0). Similarly, none of the REMS assessment plans contained measures of the Outer Setting or Inner Setting. Notably, the Shared System REMS for emtricitabine tenofovir disoproxil fumarate contained the highest proportion of measures assessing Characteristics of Individuals, with this construct accounting for 87.5% of all its assessment measures. Across all Shared System REMS, Characteristics of Individuals was assessed at a median of 7 (range 1–8) measures. Finally, the median number of Process assessment measures was 16 (range 1–57) amongst Shared System plans.

Applied to the Assessment Guidance, Knowledge and Beliefs about the Intervention and Individual Stage of Change mapped to Knowledge and Safe Use Behaviors (Fig. 1**)**. The Process construct of Engaging mapped to Outreach and Communications, while both Executing and Reflecting and Evaluating mapped to Program Implementation and Operations. Regarding the Program Implementation and Operations constructs, Executing is akin to the descriptive analysis, or descriptions of the aspects of the REMS implementation process, while Reflecting and Evaluating refer to the evaluative and causal analysis, or the corrective and preventive actions in REMS and the determination of the reasoning behind certain implementation processes.

## Discussion

To our knowledge, this is the first systematic comparative content analysis of several leading implementation science frameworks and their relative applicability and feasibility for nationally-regulated pharmaceutical risk minimization program assessment. Our evaluation found three established implementation science frameworks to be pragmatic in utility, making application to REMS programs relatively intuitive.

For example, RE-AIM considerations for evaluating health promotion programs and policies fit well with the need to evaluate REMS program recipients (like prescribers) who are agents for delivering the program to others (like patients). Application of the PROCEED constructs was also straightforward, especially with Implementation and Outcomes Evaluation, which directly matched REMS assessment categories. Overall, all three frameworks had very strong congruence with REMS assessment items, with at least 95% of items mapping to a single construct for each of the frameworks.

### Comparison of RE-AIM, PRECEDE-PROCEED, and CFIR for evaluating REMS programs

Our application of implementation science to the Assessment Guidance found that most REMS assessment measures for programs approved between 2014 and 2018 fell under Implementation and Operations, which is reasonable given FDA’s authority to require REMS and its experiences reviewing REMS implementation by the sponsors. Additionally, process data are understood to be more sensitive measures of the quality of a program than outcome data, as a poor outcome does not always occur as a result of an error in the provision of care [28]. Our analysis also found less emphasis on REMS assessment measures related to Health Outcomes. This may be because the FDA relies on good pharmacovigilance practices and pharmacoepidemiologic assessment, versus the REMS assessment itself, to collect information on safety events [29]. Interestingly, a Shared System REMS was more likely to include measures related to Health Outcomes or Safe Use Behaviors and Knowledge. This may be explained by the fact that Shared System REMS reflect sustaining programs, where processes had been implemented for some time, allowing for more opportunity to evaluate sustained effectiveness on population health outcomes.

Table 7 compares and contrasts the key strengths and limitations of RE-AIM, PRECEDE-PROCEED, and CFIR as applied to REMS programs. Based on our assessment, no single unifying framework is likely applicable for all FDA-mandated programs. Rather, each framework merits relative scientific strengths and selection should be tailored based on the need of the specific program. The evaluation also identified opportunities to further strengthen REMS program evaluation, including measures to assess institutional implementation climate (from CFIR); measures to assess the representativeness of program participation and sustainability (from RE-AIM); and measures to assess health care provider and patient values and preferences (from PRECEDE-PROCEED). The following describes the relative strengths and limitations in greater detail:

**Table 7:** Comparison of implementation science frameworks as applied to REMS program assessment

Framework	Strengths	Limitations
RE-AIM	▪ Flexible and intuitively generalizable to REMS program evaluation; has been applied across a wide range of clinical, public health, and educational settings. ▪ Includes assessment of program sustainability, which enables constructs assessing burden on the healthcare delivery system and barriers to patient access	▪ Requires careful delineation of adoption (i.e., among REMS health care implementers) vs. reach (i.e., among patients receiving the drug and thereby the benefits of the risk mitigation measures) ▪ Traditionally, an evaluation tool, although it has been used to inform program design
PRECEDE-PROCEED	▪ Explicitly links program design assumptions with program evaluation metrics ▪ Supports continuous process improvement evaluation, which enables REMS modification and revision over time	▪ Phases narrowly defined, requiring at times broader application ▪ Traditionally, applied to population-health programs; may be less applicable for some clinical settings (e.g., provider activities in a hospital)
CFIR	▪ Integrates key constructs from multiple implementation science frameworks ▪ Includes health system organizational measures, such as, administrative and leadership endorsement and organizational readiness to change, which enables greater evaluation of program implementation and operations	▪ Less intuitive to apply to REMS program evaluation, with categories often overlapping or including repetitive constructs ▪ Focused on evaluating program implementation, lacking measures to assess health outcomes and impact ▪ Traditionally, applied to clinical quality improvement programs; may be less applicable for some non-clinical settings (e.g., patient activities at home)

#### RE-AIM

As a program evaluation framework, RE-AIM aims to “improve sustainable adoption and implementation.” [26] One strength was how simple-to-use RE-AIM was and how easily adaptable it was to a spectrum of REMS assessment measures. Another was that it considers the representativeness of patients and providers [18]. This inclusion of participants’ characteristics allows for the assessment of heterogeneity of impact, which subsequently permits evaluations of patient burden and access. Health outcomes can be better measured as suggested by RE-AIM through predetermination of objectives and their impact on the changes in knowledge and safe use behaviors. REMS assessment could be strengthened by more deliberate inclusion of the RE-AIM Maintenance construct. For example, assessment measures could evaluate the evidence for integration of REMS processes and procedures into institutional policies, attrition of healthcare providers over time, or the extent to which health outcomes are sustained as a result of patient educational training [30], generating evidence for the decision of potentially releasing a REMS. If FDA started with RE-AIM to develop their REMS assessments, there could be a more even distribution of items, with more on sustainability of health care setting processes and individual behaviors and less on fidelity to every single process measure.

#### PRECEDE-PROCEED

Although intuitive to apply, not every construct mapped perfectly to a REMS measure. Some measures mapped to multiple constructs, while others did not map to a single construct. The framework does not specifically analyze outreach to providers, although it does focus on community and/or patient needs in general. A strength of the framework is that it thoroughly considers assessment of situational factors that may affect the outcome of an intervention. PRECEDE-PROCEED suggests assessment plans could make note of questions made to the REMS call center that could shed light on the differences in the tolerance for burden between individuals and subgroups. However, it is important to note that the framework does not assess resources and therefore may not be a good model for evaluating program burden. One way to strengthen the application of PRECEDE-PROCEED for REMS is to create subconstructs within PROCEED constructs as we did and assign each by participant; for example, subconstruct 7B of the Process Evaluation construct would apply only to healthcare provider measures, while subconstruct 7C also of the Process Evaluation construct would apply to patients.

#### CFIR

Our application of CFIR to REMS assessment found the framework to “open[] the ‘black box’ of … implementation” and be fruitful in evaluating implementation progress in a clinical setting, as the authors intended [31]. We were able to select constructs most relevant for REMS assessments, namely those falling under Process and Characteristics of Individuals. Some of these measures involved Reflecting and Evaluating on program implementation to improve processes. CFIR can also be used as a guide to formative evaluations for FDA to improve REMS assessments by considering constructs under Intervention Characteristics, the Outer Setting, and the Inner Setting. Through its 39 different subconstructs, CFIR can also inform the design and conduct of REMS. For example, on the construct of Intervention Characteristics, Adaptability can be measured in the earlier REMS assessment plans to evaluate the degree to which REMS requirements must be tailored and refined to meet local needs and sponsor capacities. In the Outer Setting, Patient Needs and Resources can be assessed to the extent that the REMS program is patient-focused and addresses patient barriers. Measures of participants’ knowledge about the REMS-related risk or of any safe use conditions that are needed to mitigate the risk can be aided by assessment of the participant’s values and attitudes towards that knowledge or safe use behavior. Participants’ Readiness for Implementation including physical resources, leadership engagement, and culture can also be assessed under the Inner Setting. Validating design assumptions would then allow for more thoughtful modifications and ultimately improve program performance. If FDA develops REMS assessment measures starting from CFIR, there would be considerations about the design aspects of the REMS characteristics, thoughts about how the REMS would fit into the broader healthcare setting, and participants’ receptiveness about the REMS.

### Implications for regulated risk management plans

The use of frameworks offers several advantages to advance the science of pharmaceutical risk minimization program evaluation. First, grounding assessment plans through frameworks encourages consistency, standardization, and completeness in evaluating REMS to facilitate cross program comparisons and foster generalizable knowledge. Second, frameworks are valuable for building the evidence base for synthesizing implementation strategies to improve outcomes. They can be used to drive data towards more meaningful information and increase the likelihood that REMS design, implementation, and outcomes are effective. Currently, setting, context and implementation strategy selection are under-reported in published evaluations making it difficult to compare effectiveness [32]. Conceptual models have been proposed for developing efficient strategies for the measurement of the effectiveness of European Medicines Agency RMMs [33]. Like REMS, RMMs can also benefit from the application of implementation science in the aspects of the delivery context, attributes of the proposed intervention, and characteristics of the intended adopters [15].

Although the present evaluation focused on using implementation science frameworks for REMS assessment, these frameworks can also be used to inform REMS design. For example, the goal of the Addyi REMS program is to mitigate the increased risk of syncope and hypotension associated with drinking while taking the medication. Because patients’ current and past drinking behavior must be assessed before Addyi is prescribed, a framework focused on changing health behavior might be most suitable [34]. PRECEDE-PROCEED considers the spectrum of behavior change from the predisposing, enabling, and reinforcing factors to the impact of the program on these factors and could therefore inform a REMS program like Addyi that is designed to change behavior. REMS programs with health education as its main intent, such as the Opioid Analgesic REMS, could benefit from using RE-AIM as this framework not only poses questions related to assessing individuals reached and the immediate and long-term effectiveness of the knowledge transfer but also how the setting and operations could facilitate or impede the change in knowledge [35]. Finally, as CFIR focuses on implementing evidence-based interventions with demonstrated effectiveness, it is a suitable framework for a Shared System REMS such as that of bosentan where the goal is to sustain implementation effectiveness as new drugs or generics enter the Shared System REMS [36].

Lastly, under FDA’s PDUFA V commitments to modernize post-marketing drug safety evaluation, it has committed to efforts on implementing a structured benefit-risk assessment process [37]. The benefit-risk assessment framework has identified “Risk and Risk Management” as one explicit dimension, with an area of interest concerning how information on evidence and uncertainties can be communicated to the public. Implementation science frameworks can be used to ensure that risk management uncertainties are systematically evaluated and categorized based on factors known to affect real-world implementation of health programs. In turn, this level of rigor in a policy context facilitates the selection of interventions with clinical benefit for healthcare organizational settings to ultimately improve patient health.

## Conclusions

As FDA considers feedback from stakeholders and public comments in its finalization of the Assessment Guidance, this research can serve as one source of input. This research demonstrates the feasibility of implementation science frameworks to be applied to REMS assessment plans. Frameworks such as RE-AIM, PRECEDE-PROCEED, and CFIR provide a logical, structured approach for determining what should be measured, when they should be measured, and the process and impact indicators for facilitating these measurements. Application of implementation science to REMS assessment measures reveals a need to consider the design and sustainability of REMS programs in the assessment plans. Future REMS assessment plans can consider an element from each of the three frameworks adapted, including patient values and preferences, representativeness, and the implementation climate. Using this information, sponsors can evaluate matters of stakeholder interest, such as patient burden and access through assessment of heterogeneity. Burdens on patients and the healthcare system can be further reduced by determining core explanatory measures for every step of the evaluation continuum to prevent unnecessary interventions when one core primary measure would be sufficient for determining whether the REMS goal could be met.

## Supplementary Information

**Additional file 1.** Examples of search strings used to identify frameworks for assessing REMS programs.

**Additional file 2.** Assessment Guidance categories mapped to framework constructs.

**Additional file 3.** Flow diagram of 2014–2018 active REMS with ETASU program selection for content analysis of assessment plans.

## Data Availability

The data that support the findings of this study are available from the corresponding author upon reasonable request.

## Abbreviations

ANDA: Abbreviated new drug application
BLA: Biologic license application
CFIR: Consolidated framework for implementation research
CP: Communication plan
ETASU: Elements to assure safe use
FDA: Food and drug administration
MG: Medication guide
NDA: New drug application
PDUFA: Prescription drug user fee act
PRECEDE-PROCEED: Predisposing, reinforcing, and enabling constructs in educational diagnosis and evaluation – policy, regulatory, organizational, construct in, educational and environmental, development
RE-AIM: Reach effectiveness adoption implementation maintenance
REMS: Risk evaluation and mitigation strategies
RMM: Risk minimization measures
