# Supplementary material for: Pragmatic applications of implementation science frameworks to regulatory science: an assessment of FDA Risk Evaluation and Mitigation Strategies (REMS) (2014–2018)
Source: BMC Health Serv Res. 2021 Aug 6;21:779. doi: 10.1186/s12913-021-06808-3 (PMC8348874; doi:10.1186/s12913-021-06808-3)
Supplement: Supplementary file 1 — Additional file 1. Examples of search strings used to identify frameworks for assessing REMS programs. [file 12913_2021_6808_MOESM1_ESM.docx]

**Additional file 1** Examples of search strings used to identify frameworks for assessing REMS programs

| REMS search strings | Dissemination and implementation search strings |
| --- | --- |
| “Risk evaluation and mitigation strategies” OR “REMS” | “implementation” OR “framework” or “implementation framework” |
| “Risk evaluation and mitigation strategies” OR “REMS” | “RE-AIM” |
| “Risk evaluation and mitigation strategies” OR “REMS” | “PRECEDE-PROCEED” |
| “Risk evaluation and mitigation strategies” OR “REMS” | “CFIR” OR “Consolidated Framework for Implementation Research” |
