# Supplementary material for: Pragmatic applications of implementation science frameworks to regulatory science: an assessment of FDA Risk Evaluation and Mitigation Strategies (REMS) (2014–2018)
Source: BMC Health Serv Res. 2021 Aug 6;21:779. doi: 10.1186/s12913-021-06808-3 (PMC8348874; doi:10.1186/s12913-021-06808-3)
Supplement: Supplementary file 2 — Additional file 2. Assessment Guidance categories mapped to framework constructs. [file 12913_2021_6808_MOESM2_ESM.docx]

**Additional file 2** Assessment Guidance categories mapped to framework constructs

| **FDA Assessment Categories** | **Implementation Science Frameworks** | | |
| --- | --- | --- | --- |
|  | **RE-AIM** | **PRECEDE-PROCEED** | **CFIR** |
| **Design*** | Not Discussed | PRECEDE (Phases 1-4) | Intervention Characteristics, Outer Setting, Inner Setting (Constructs I-III)  Process – Planning (V.A) |
| **Outreach and Communications** | Reach, Adoption | Implementation | Process – Engaging (V.B) |
| **Implementation and Operations** | Implementation | Process Evaluation | Process – Executing (V.C)  Reflecting and Evaluating (V.D) |
| **Safe Use Behaviors and Knowledge** | Effectiveness, Implementation | Impact Evaluation | Characteristics of Individuals – Knowledge and Beliefs about the Intervention (IV.A), Individual Stage of Change (IV.C) |
| **Health Outcomes** | Effectiveness | Outcome Evaluation | Not Discussed |
| **Sustainability*** | Maintenance | Not Discussed | Not Discussed |

*Categories not included in the Assessment Guidance
