# Supplementary material for: Pragmatic applications of implementation science frameworks to regulatory science: an assessment of FDA Risk Evaluation and Mitigation Strategies (REMS) (2014–2018)
Source: BMC Health Serv Res. 2021 Aug 6;21:779. doi: 10.1186/s12913-021-06808-3 (PMC8348874; doi:10.1186/s12913-021-06808-3)
Supplement: Supplementary file 3 — Additional file 3. Flow diagram of 2014–2018 active REMS with ETASU program selection for content analysis of assessment plans. [file 12913_2021_6808_MOESM3_ESM.doc]

**Additional file 3** Flow diagram of 2014-2018 active REMS with ETASU program selection for content analysis of assessment plans

REMS approved between 1/1/2014 – 12/31/2018 assessed for eligibility (n=46)

Excluded released REMS (n=3)

Active REMS (n=43)

Excluded REMS with CPs or MGs as the only elements (n=20)

Analyzed (n=23)

^a^ CP, Communication Plan, MG, Medication Guide

^b^ REMS programs were accessed for eligibility January 2019
